# Disulfiram targeting lymphoid malignant cell lines via ROS-JNK activation as well as Nrf2 and NF-kB pathway inhibition

**DOI:** 10.1186/1479-5876-12-163

**Published:** 2014-06-11

**Authors:** Jie Zha, Feili Chen, Huijuan Dong, Pengcheng Shi, Yao Yao, Yanyan Zhang, Rongwei Li, Shiyun Wang, Peng Li, Weiguang Wang, Bing Xu

**Affiliations:** 1 Department of Hematology, Nanfang Hospital, Southern Medical University, Guangzhou 510515, China; 2 Key Laboratory of Regenerative Biology, South China Institute for Stem Cell Biology and Regenerative Medicine, Guangzhou Institutes of Biomedicine and Health, Chinese Academy of Sciences, Guangzhou 510530, China; 3 Guangdong Provincial Key Laboratory of Stem Cell and Regenerative Medicine, South China Institute for Stem Cell Biology and Regenerative Medicine, Guangzhou Institutes of Biomedicine and Health, Chinese Academy of Sciences, Guangzhou 510530, China; 4 Drug Discovery Pipeline, Guangzhou Institutes of Biomedicine and Health, Chinese Academy of Sciences, Guangzhou 510530, China; 5 Research Institute in Healthcare Science, Faculty of Science and Engineering, University of Wolverhampton, Wolverhampton, UK

**Keywords:** Disulfiram, Nrf2, NF-κB, JNK, Apoptosis

## Abstract

**Background:**

Disulfiram (DS), an anti-alcoholism drug, demonstrates strong antitumor activity in a copper (Cu)-dependent manner. This study investigates the cytotoxicity of DS/Cu complex in lymphoid malignant cell lines *in vitro* and *in vivo*.

**Method:**

Raji cells were subjected to different treatments and thereafter MTT assay, flow cytometry were used to determine IC_50_ and apoptotic status. We also tested the cytotoxicity of DS/Cu in acute lymphoblastic leukemia cell line Molt4 *in vitro. In vivo* experiments were also performed to demonstrate the anticancer efficacy of DS/Cu in Raji cells xenografted nude mice.

**Results:**

In combination with a low concentration (1 μM) of Cu^2+^, DS induced cytotoxicity in Raji cells with an IC_50_ of 0.085 ± 0.015 μM and in Molt4 cells with an IC_50_ of 0.435 ± 0.109 μM. The results of our animal experiments also showed that the mean tumor volume in DS/Cu-treated mice was significantly smaller than that in DS or control group, indicating that DS/Cu inhibits the proliferation of Raji cells *in vivo*. DS/Cu also induced apoptosis in 2 lymphoid malignant cell lines. After exposure to DS (3.3 μM)/Cu (1 μM) for 24 hours, apoptosis was detected in 81.03 ± 7.91% of Raji cells. DS/Cu induced significant apoptosis in a concentration-dependent manner with the highest apoptotic proportion (DS/Cu: 89.867 ± 4.69%) at a concentration of 2 μM in Molt4 cells. After 24 h exposure, DS/Cu inhibits Nrf2 expression. Flow cytometric analysis shows that DS/Cu induced ROS generation. DS/Cu induced phosphorylation of JNK and inhibits p65 expression as well as Nrf2 expression both *in vitro* and *in vivo*. N-acetyl-L-cysteine (NAC), an antioxidant, can partially attenuate DS/Cu complex-induced apoptosis and block JNK activation *in vitro*. In addition, NAC is able to restore Nrf2 nuclear translocation and p65 expression.

**Conclusion:**

Our study manifests that DS/Cu complex targets lymphoid malignant cells *in vitro* and *in vivo*. Generation of ROS might be one of core steps in DS/Cu induced apoptosis. Moreover, ROS-related activation of JNK pathway and inhibition of NF-κB and Nrf2 may also contribute to the DS/Cu induced apoptosis.

## Introduction

Lymphoid malignances are tumors of the immune system. Although treatments including chemotherapy and stem cell transplantation are developing very fast, patients still suffer from relapse and treatment related complications. In order to improve the prognosis and life quality of patients with lymphoid malignances, new therapeutic strategies are urgently demanded.

Disulfiram (DS) is an anti-alcholism drug used in clinic for over 60 years [1]. DS belongs to dithiocarbamate family which is able to strongly chelate Cu and forms disulfiram/cooper (DS/Cu) complex. DS/Cu complex is highly cytotoxic to many solid tumors while DS or Cu alone had few anti-tumor effects [2-4]. However, the effect of DS/Cu on lymphoid malignancies has not been reported yet.

Reactive oxygen species (ROS) contains a group of oxygen-containing chemical species normally generated from mitochondrial respiratory chain reaction with reactive chemical properties [2]. Cancer cells usually possess and tolerate higher ROS activity than normal cells [3]. It has been demonstrated that further increasing ROS exposure induced by ROS-generating agents such as DS/Cu [3] can exhaust the cellular antioxidant capacity and thus induce apoptosis in tumor cells.

Nrf2 is a transcription factor with strong antioxidant effect which protects cancer cells from the damages induced by ROS, anticancer drugs and other harmful chemicals [3]. Previous publications have demonstrated the causal relationship between Nrf2 and chemo-resistance [5-7]. So, simultaneous down-regulation of Nrf2 and induction of ROS in cancer cells can lead to more apoptosis.

ROS-induced apoptosis is also highly reliant on persistent activation of pro-apoptotic mitogen activated protein kinase (MAPK) pathways [8]. C-Jun NH2-terminal kinase (JNK) is an important member of the MAPK family [9]. Our previous study has already shown that activation of JNK can sensitize resistant cells to anticancer drugs indicating the important role of JNK in drug-induced apoptosis [10].

NF-κB is one of the major chemo-resistance-related anti-apoptotic factors [8]. Lymphoid malignant cells possess high levels of constitutive NF-κB activity, leading to resistance to apoptosis [7]. Thus, NF-κB is an attractive molecular target for therapeutic intervention. Previous studies have also shown close interactions between ROS and NF-κB [8].

Although ROS could trigger apoptosis in cancer cells, the ROS-induced anti-apoptotic factors, e.g. NF-κB and Nrf2, can counteract apoptotic effects of ROS. Hence, development of drugs that can simultaneously activate sustained ROS pro-apoptotic pathway and inhibit NF-κB and Nrf2 activity may improve cancer chemotherapy. This study demonstrates that DS/Cu simultaneously activates ROS-JNK pro-apoptotic pathway and down-regulates NF-κB and Nrf2 anti-apoptotic pathways. Therefore, DS/Cu shows strong anti-cancer efficacy *in vitro* and *in vivo*.

## Materials and methods

### Cell lines and experimental animals

Raji and Molt4 cell lines were cultured at 37°C in 5% CO_2_ in RPMI 1640 with 10% heat-inactivated FBS, 100 units/mL penicillin and 100 μg/mL streptomycin. The BALB/C nude mice (4–5 weeks of age, non-fertile, female and 18–20 g each) were purchased from the Experimental Center of Southern Medical University. All animal study procedures were approved by the Southern Medical University Animal Care and Use Committee.

### Establishment of non-Hodgkin lymphoma animal model and treatment

Mice were injected subcutaneously on the back with 1×10^7^ Raji cells suspended in 0.2 mL sterile PBS. Nude mice were randomly divided into 3 groups, and with 6 mice in each group. The day mice received cell injection was counted as day 0. Mice in the control group were administrated with normal saline by oral gavage once per day for 10 days from +8 day to +12 day and +15 day to +19 day while those in DS and DS/Cu groups were given DS (2.88 mg/20 g per morning) and DS/Cu (DS: 2.88 mg/20 g per morning, Cu: 0.012 mg/20 g per afternoon) respectively. The dosage used here was determined by our preliminary experiment. Length and width of the tumor were measured with a vernier caliper from the beginning of treatment every other day with the volume being calculated using the formula V = a × b^2^/2 and growth curve being documented. The weight of each mouse was also recorded every other day. Mice were sacrificed on day 20, and the tumors were removed for western blotting analysis.

### MTT cytotoxicity assay

Drug cytotoxicity was determined using the colorimetric MTT assay. Briefly, cells (1 × 10^5^ cells/well) were plated into 96-well plates containing 100 μl of the growth medium in the absence or presence of increasing concentrations of drugs at 37°C in 5% CO_2_ for 24 h and 48 h, MTT (50 μl/well, 5 mg/ml in PBS) was then added and incubated for 4 h at 37°C. The cells were further treated with 100 μl DMSO to dissolve the dark blue crystals of formazan and the absorbance was measured at 570 nm in a microplate reader (ELX800, BioTEK, USA). All experiments were repeated at least three times with triplicate in each experiment. The cytotoxicity of DS or DS/Cu was analyzed and concentration-effect curves were generated as a plot of the fraction of affected cells versus drug concentration. Growth inhibition was expressed as a percentage of the untreated controls that were processed simultaneously. The IC_50_ was defined as the concentration that inhibited cell growth by 50% (50% reduction of absorbance) compared with untreated controls.

### Flow cytometric analysis of apoptotic cells

Cells (3 × 10^5^) cultured in 25 cm^2^ flasks and exposed to different treatments for 6 h, 12 h, or 24 h were harvested respectively, washed twice with ice-cold PBS and then re-suspended in 500 μl binding buffer. The cells were further incubated with Annexin V-FITC and Propidium Iodide for 15 min at room temperature in the dark according to the manufacturer’s instructions. The stained cells were analyzed by flow cytometry using FACS Calibur (BD Biosciences, Oxford, UK) and Cell Quest (BD Biosciences) software.

### Determination of ROS production

ROS production in cells was determined utilizing 2’,7’- dichlorodi hydro fluorescein diacetate (DCFDA). Briefly, 4 × 10^5^/ml cells were taken in a culture dish and treated with DS or DS/Cu for 6 h, 12 h or 24 h. After the treatment, cells were collected and DCFDA (Sigma-Aldrich, Dorset, UK) was added to the cell suspension at a final concentration of 10 μM. After 30 minutes of incubation in the dark at 37°C, cells were centrifuged and the pellet was washed twice with ice-cold PBS. The pellet was then resuspended in FACS buffer and the fluorescence was analyzed with FACS Calibur (BD Biosciences, Oxford, UK) and CellQuest (BD Biosciences) software. DCFDA fluorescence intensity was measured in FL-1 with an excitation wavelength of 488 nm and an emission wavelength of 530 nm. The percentage of ROS producing cells was calculated by counting only those cells, which produced high levels of ROS.

### Western blot analysis

Whole protein (50 μg/lane) from each sample was resolved in 10% SDS-polyacrylamide gel electrophoresis (PAGE), transferred to a PVDF membrane (Millipore, UK) and blotted with various antibodies. Non-specific binding was avoided by blocking the nitrocellulose membrane with 5% skimmed milk in TBS-T for 1 h. The 5% skimmed milk in TBS-T was also used to dilute primary (SAPK/JNK, rabbit polyclonal, 1:1000, CST; Phospho-SAPK/JNK, rabbit polyclonal 1:1000, Cell Signaling techonology; Phospho-c-jun, rabbit polyclonal, 1:500, Bioworld Technology Co., Ltd., c-jun, rabbit polyclonal, 1:1000, Santa cruz; P65, rabbit polyclonal, 1:1000, Santa Cruz; Nrf2, rabbit polyclonal, 1:1000) and HRP-conjugated monoclonal secondary (1:5000; Amersham Pharmacia Biotech, NJ) antibodies. The membranes were incubated with the primary antibodies overnight at 4°C and in the secondary antibody for 1 h at room temperature. The quantity of protein loaded was verified by staining the same membranes with anti-β actin antibody (1:2000, Sigma-Aldrich, Dorset, UK). The signals were detected on X-ray films using an ECL Western blotting detection kit (Amersham, Pharmacia Biotech).

### Statistical analysis

All results were analyzed by Student’s t-test and ANOVA using SPSS 13.0. The statistical significance was indicated by a p value < 0.05.

## Results

### The cytotoxicity of DS in both Raji and Molt4 cells was Cu-dependent

**Figure 1:**
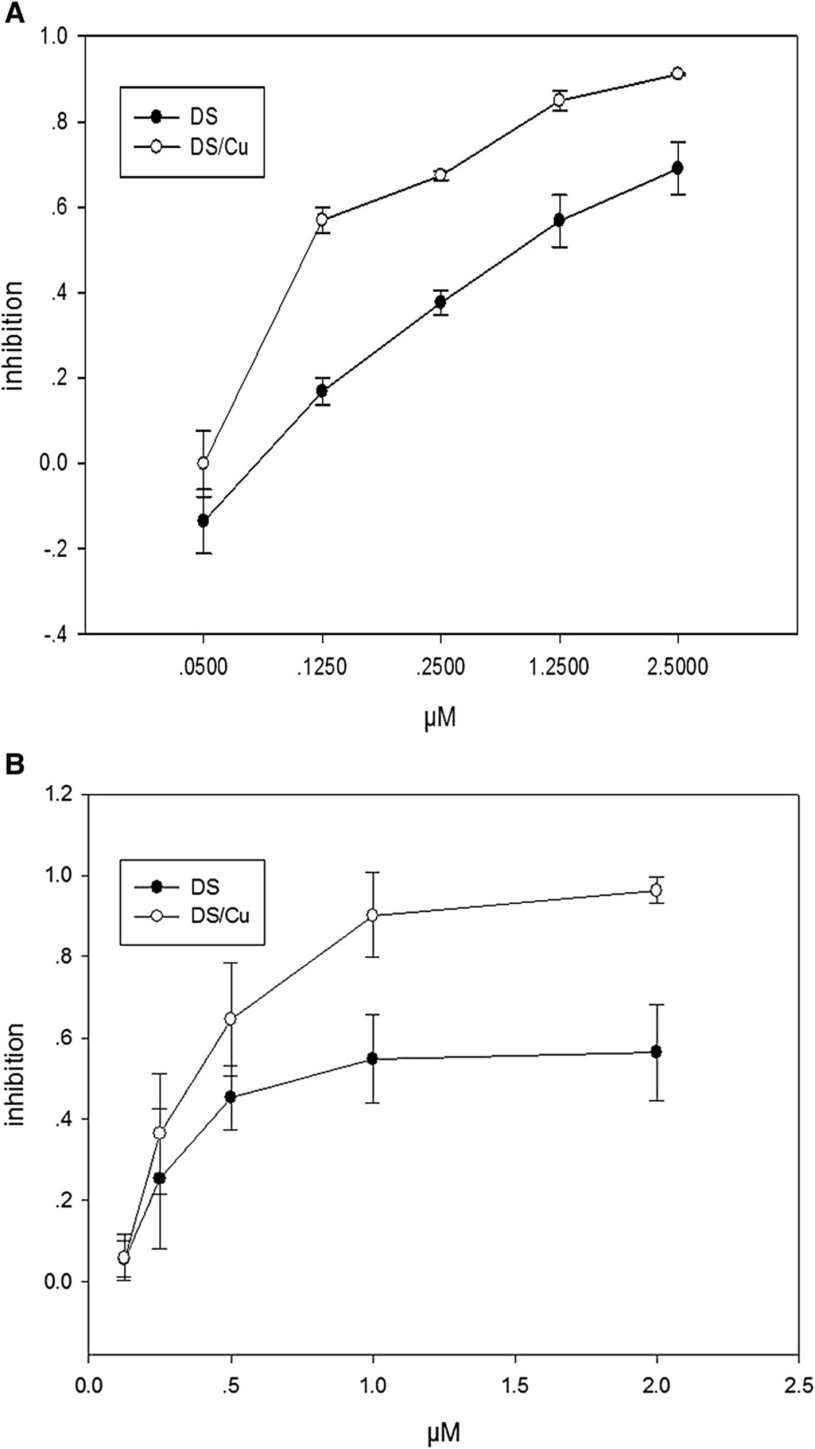
**The cytotoxicity of DS in Raji and Molt4 cells was Cu-dependent. (A)** MTT results of DS and DS/Cu in Raji cells at 72 h (n = 3). **(B)** MTT results of DS and DS/Cu in Molt-4 cells at 24 h (n = 3).

Cytotoxicity of DS or DS/Cu was determined using MTT assay. In CuCl_2_ (1 μM)-supplemented medium, DS was highly cytotoxic to Raji cell lines (IC_50_72h_: 0.085 ± 0.015 μM; Figure 1A). DS was also toxic to cancer cell lines in the complete medium without CuCl_2_ supplement with higher IC_50_ (IC_50_72h_:0.793 ± 0.08 μM; *p* <0.001 Figure 1A). To further confirm the cytotoxicity of DS or DS/Cu to lymphocyte derived tumor, we tested another type of tumor cell line Molt4 *in vitro*. As shown in Figure 1B, DS/Cu was highly cytotoxic to Molt4 cells with IC_50_24h_ = 0.435 ± 0.109 μM. DS alone was also toxic with higher IC_50_ of 1.314 ± 0.229 μM in Molt4 cells.

**Figure 2:**
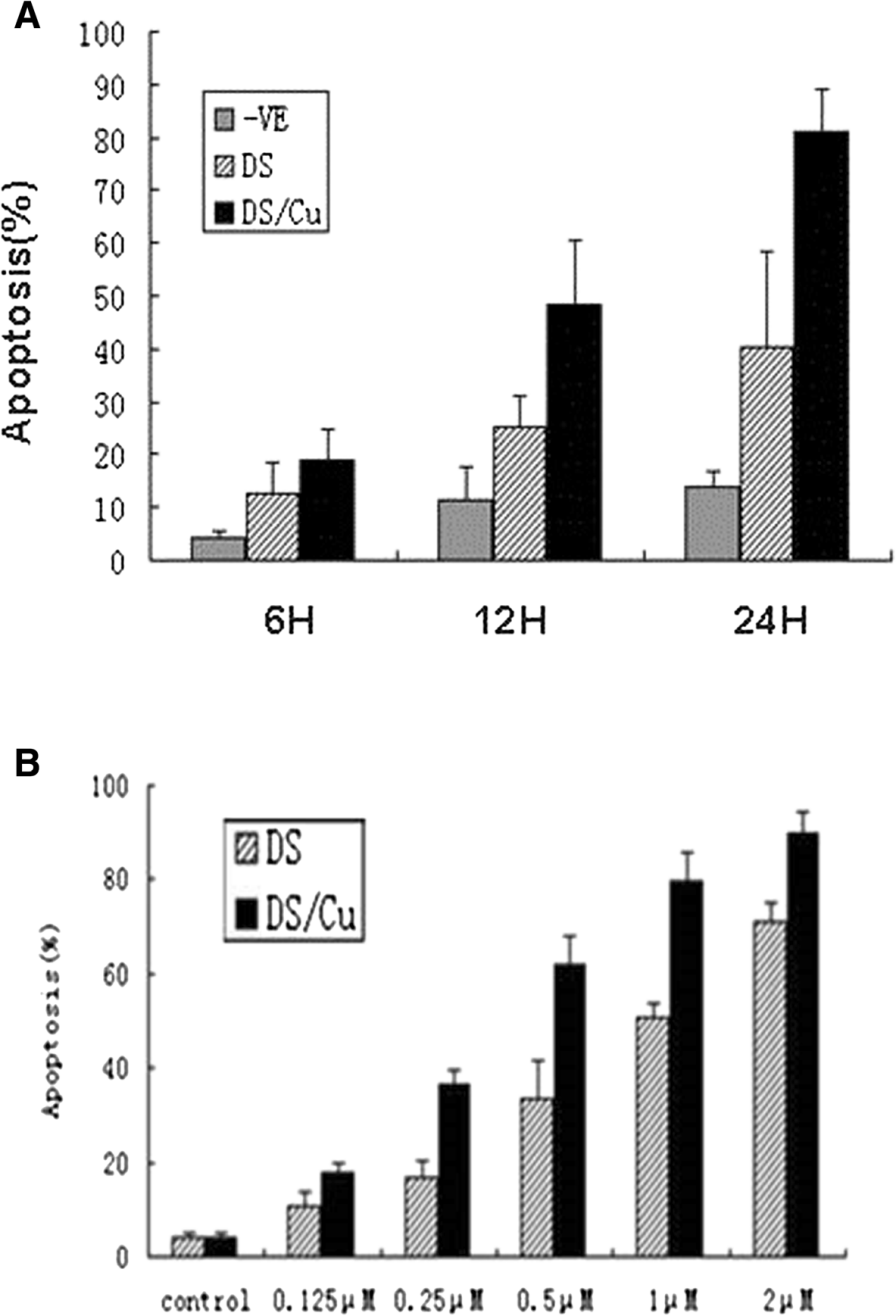
**DS with or without Cu induces apoptosis in lymphoid malignant cells. (A)** The apoptotic Raji cells was measured by flow cytometry after treated with disulfiram with or without Cucl2 at different time points ( 6 h,12 h,24 h) (n = 3). **(B)** Molt-4 cells were treated with different combination of disulfiram and Cucl2, after which the apoptotic cells were determined by flow cytometry (n = 3).

Furthermore, apoptosis of Raji cells being treated with DS at the concentration of IC_50_24h_ (3.3 μM) with or without Cu (1 μM) for 6, 12 and 24 h was analyzed using Annexin V-FITC/PI staining method (Figure 2A). A higher apoptotic rate was achieved at any time-point treated by DS/Cu (P < 0.05; P < 0.01; P < 0.01). It was also observed that the apoptotic rate of cells treated by DS/Cu was time-dependent with a higher apoptotic ratio at 24 h than that at 6 h and 12 h (P < 0.001). In addition, Molt4 cells treated with DS at different concentrations (0.125, 0.25, 0.5, 1, 2 μM) in combination with Cu (1 μM) or without Cu for 24 h were subjected for apoptotic analysis. Except for 0.125 μM DS alone, DS with or without Cu could induce significant apoptosis in a concentration-dependent manner with the maximal apoptotic proportion (DS: 70.943 ± 3.987, DS/Cu: 89.867 ± 4.69%) at a concentration of 2 μM. Interestingly, the apoptosis rate of Molt4 cells that were treated by DS/Cu was higher (Figure 2B).

### DS/Cu significantly increases the ROS level in Raji cells

**Figure 3:**
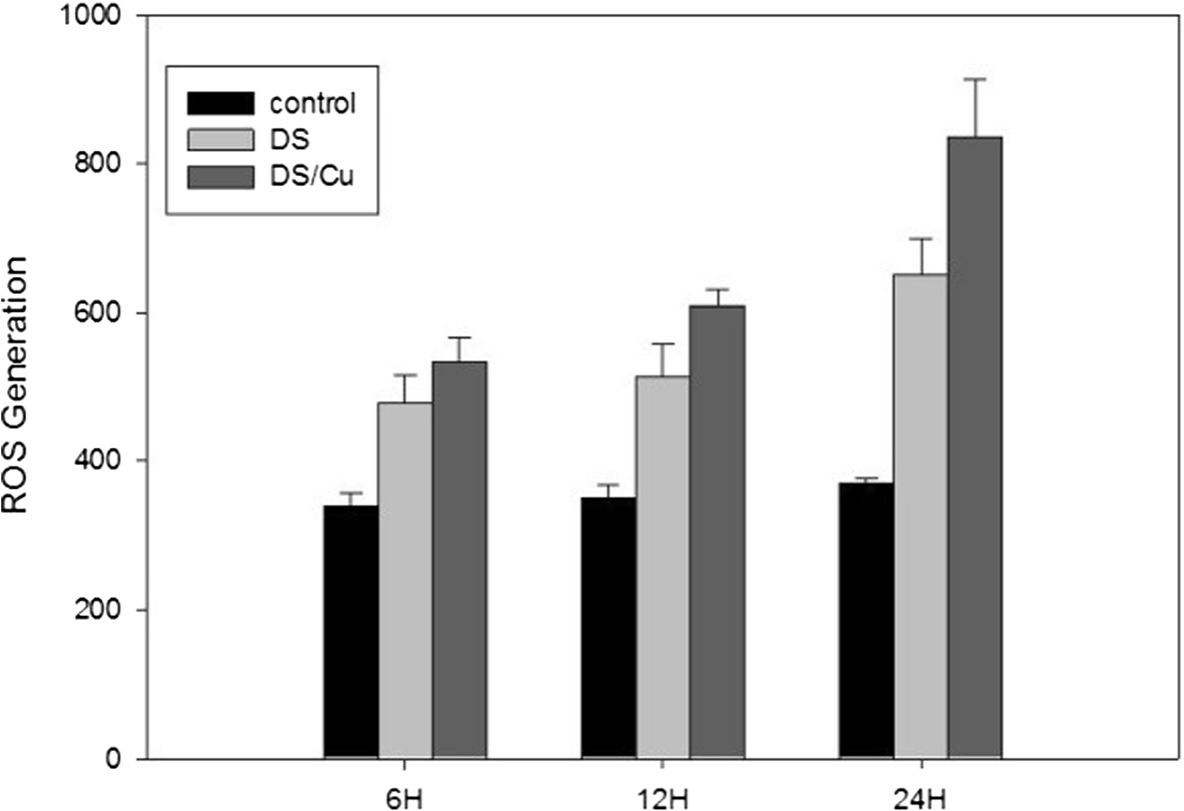
**DS or DS/Cu could induce ROS activity in Raji cells in a time-dependent manner.** ROS level of Raji cells after exposure to DS (3.3 μM) with or without Cu (1 μM) for 6 h, 12 h, and 24 h.

In accordance with previous studies, our experiment demonstrates that ROS activation is induced by DS/Cu [11]. As shown in Figure 3, ROS levels were detected after Raji cells being treated with DS (3.3 μM) or DS/Cu (DS:3.3 μM;Cu:1.0 μM) at 6 h, 12 h, and 24 h . Both DS and DS/Cu can increase the ROS activity in a time-dependent manner (DS vs control :P < 0.01; DS/Cu vs control: P < 0.01). However, DS/Cu significantly induces ROS activity in Raji cells compared with DS alone, and the highest ROS activity can be detected in cells treated with DS/Cu at 24 h(DS vs DS/Cu: 6 h: P < 0.01; 12 h: P < 0.001; 24 h: P < 0.001).

### DS/Cu significantly influences the expression of Nrf2 in Raji cells

**Figure 4:**
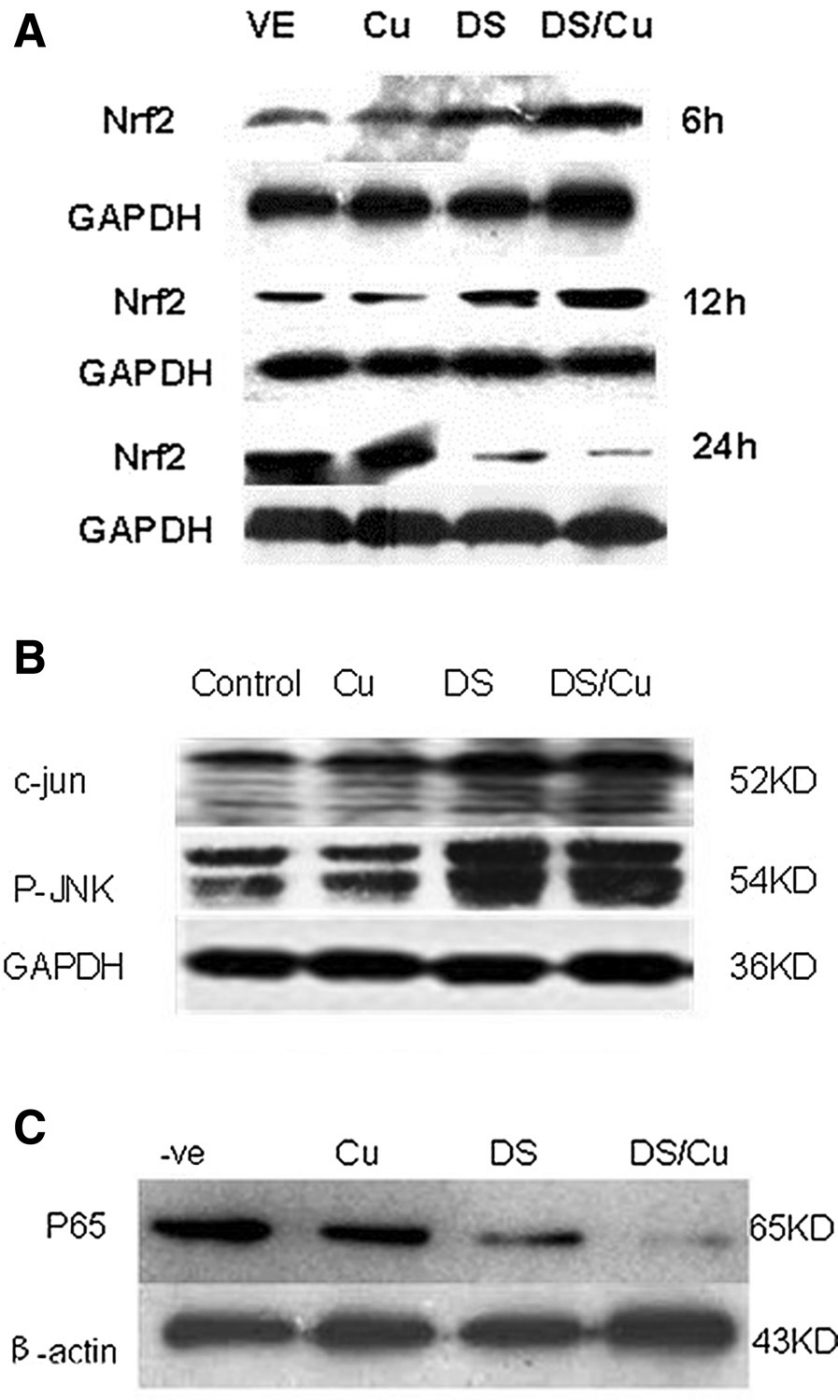
**The effect of DS/Cu on the expression level of Nrf2 as well as on JNK and NF-κB pathways.** Raji cells were exposed to DS_3.3μM_/Cu_1 μM_ , DS _3.3μM_ or Cu _1μM_ for indicated time lengths. The expression levels and phosphorylation status of proteins in JNK **(B)** (after 6 hours’ treatment) as well as the expression levels of Nrf2 **(A)** (after 24 hours’ treatment) and NF-κB pathways **(C)** (after 24 hours’ treatment) were detected by western blot. All the proteins detected here were from whole proteins. –VE: control.

Nrf2 is a transcription factor which plays a vital role in activating antioxidant response that decreases ROS [3]. It has been reported that the expression of Nrf2 is correlated with the ROS activity [6]. In line with previous report, ROS activity increased when Raji cells were treated with DS or DS/Cu for 12 h (Figure 4A). However, when Raji cells were exposed to DS or DS/Cu for a longer period of time (18 h or 24 h for example), the expression of Nrf2 decreased. The decrease of Nrf2 expression level was most obvious after having been treated with DS or DS/Cu for 24 h (Figure 4A).

### DS/Cu triggered persistent activation of JNK pathway

Figure 4B shows the effect of DS, Cu or DS/Cu on the activation of the JNK pathway. The expression of phosphorylated JNK and c-jun proteins significantly increased in Raji cells after exposure to DS (3.3 μM) with or without Cu (1.0 μM), especially in those treated with DS/Cu. However, the expression of these proteins was only mildly affected when treated with Cu (1.0 μM) alone.

### DS/Cu inhibited NF-κB activity in Raji cell lines

Previous publications have already demonstrated the close relationship between DS/Cu and NF-κB activity [8]. p65 protein is an important component of NF-κB family and its expression is correlated with NF-κB activity. Figure 4C shows that DS (3.3 μM) with or without Cu (1.0 μM) inhibits p65 protein expression. The strongest inhibition was observed in cells treated with DS/Cu.

### ROS activation was responsible for DS/Cu-induced JNK, NF-κB and Nrf2 changes

**Figure 5:**
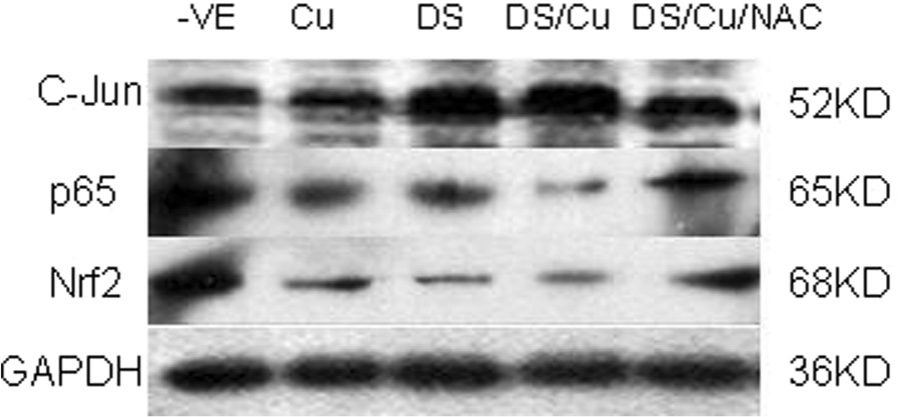
**ROS is the key mediator of DS/Cu induced JNK, NF-κB and Nrf2 changes.** The Raji cell lines were exposed to Cu_1.0μM_, DS_3.3μM_ or DS_3.3μM_/Cu_1.0μM_ in combination with NAC (10 mM) for 24 h. The expressions of c-jun, p65 and Nrf2 were detected by western blot. All the proteins detected here were from whole proteins. –VE: control.

Previous reports and the data collected from this study demonstrate that the cytotoxicity of DS is Cu-dependent [11], indicating ROS might be the mediator of DS/Cu induced JNK, NF-κB and Nrf2 changes. Figure 5 shows that when Raji cells were treated with DS (3.3 μM), Cu (1.0 μM) or DS/Cu in the presence of ROS inhibitor NAC (10 mM) for 24 h, the activation of JNK pathway, the inhibition of NF-κB activity and the decrease of Nrf2 expression level by DS/Cu were abolished.

### DS/Cu inhibited the growth of xenografts derived from Raji cells

**Figure 6:**
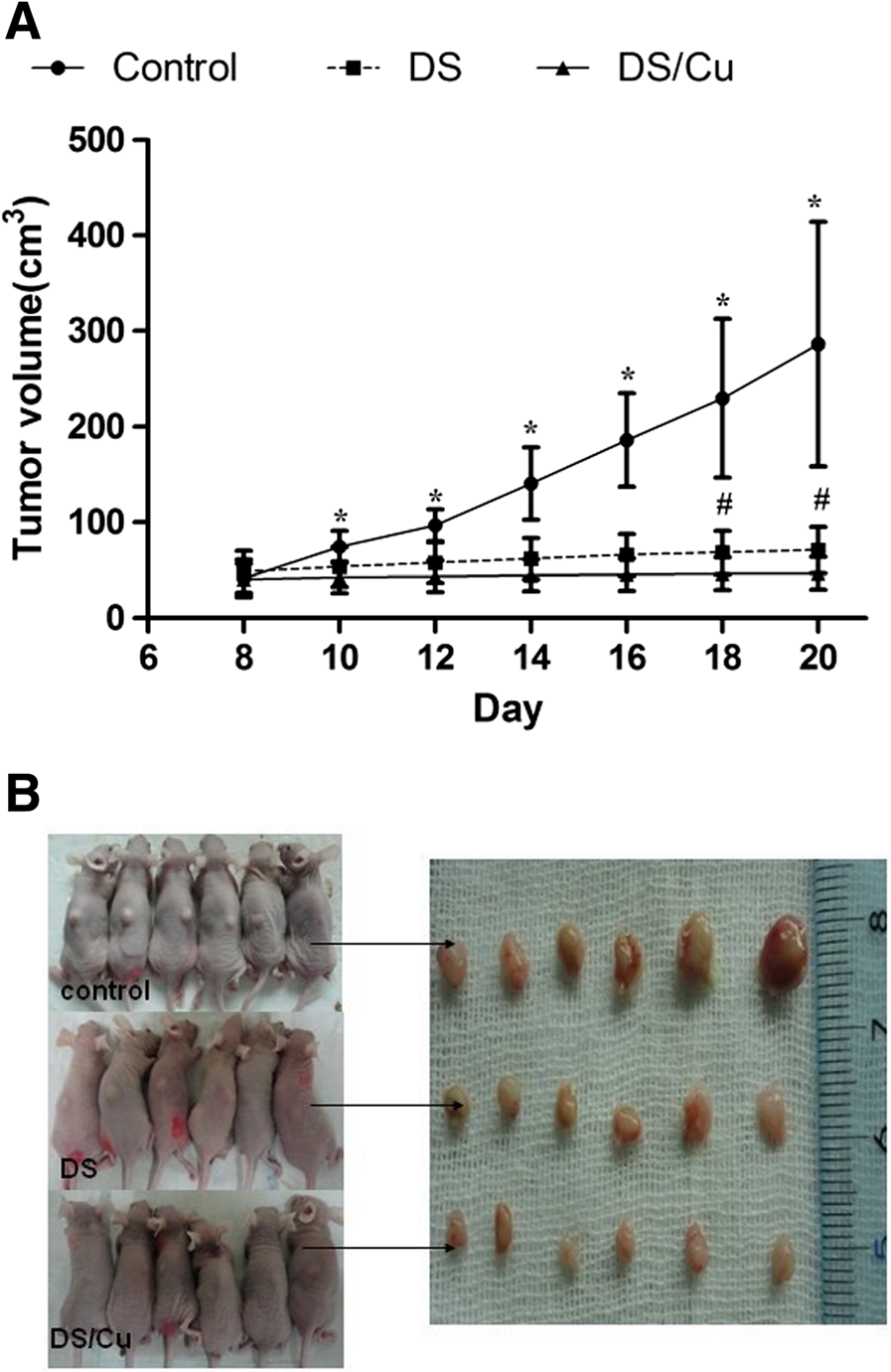
**Apoptosis of Raji cells could be induced by DS/Cu in vivo. (A)** Tumor volume growth curve. **(B)** Image of the subcutaneous lymphoma tumor. *DS/Cu v.s. Control: P < 0.05; #DS/Cu v.s. DS: P < 0.05.

To determine if DS/Cu had similar activity *in vivo*, a lymphoma mouse model was established. Mice in control, DS, and DS/Cu groups were orally given the same volume of normal saline, DS, or DS/Cu respectively for 10 days. All the mice had survived until the end of the experiment. As shown in Figure 6A and 6B, the mean tumor volume of mice in DS/Cu group was significantly smaller than DS and control group, indicating that DS/Cu inhibits the proliferation of Raji cells *in vivo* (*P* < 0.05).

### DS/Cu restored the expression of Nrf2 and P65 while activating JNK pathway in vivo

**Figure 7:**
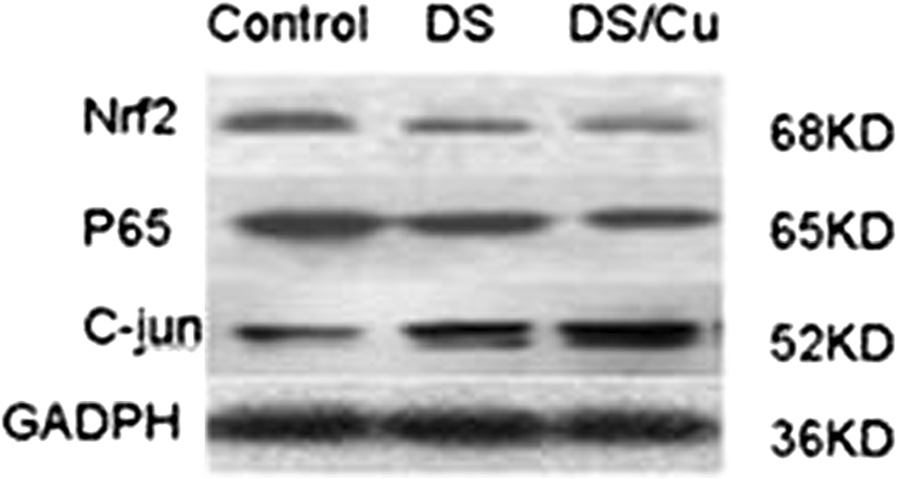
**DS/Cu restores the expression of Nrf2 and P65 while activating JNK pathway in vivo.** Representative western blots analysis of tumor tissue extracts with antibodies against Nrf2, P65 and c-jun.

Our results clearly demonstrate that DS/Cu induces the apoptosis of Raji cells through inducing ROS activity and activating JNK pathway while inhibiting p65 and Nrf2 expression in vitro. Next, we examined whether the mechanism involved might be the same *in vivo*. As shown in Figure 7, the expression of Nrf2 and P65 was reduced when Raji cells were treated with DS or DS/Cu, and the largest reduction was observed in the DS/Cu group. Persistent activation of JNK pathway was also observed in DS or DS/Cu treated groups.

## Discussion

Disulfiram is a Food and Drug Administration-approved anti-alcoholism drug used in clinic for more than 6 decades with numerous available pre-clinical and clinical data [1]. Previous studies have already demonstrated its low toxicity to normal tissues [1]. Recently, the anti-cancer ability of disulfiram has been reported [11]. Moreover, DS can facilitate intracellular Cu uptake in cancer cells and potentiate the cytotoxicity of anticancer drugs in drug resistant and sensitive breast, colon cancer and leukemia cell lines [12]. We have reported that DS/Cu is cytotoxic and able to reverse chemoresistance in drug resistant HL60/DOX leukemia cell line *in vitro*[10]. However, there was no report about the cytotoxicity and related molecular mechanisms of DS/Cu in lymphoid malignant cell lines.

In this study we first demonstrated that DS has a high cytotoxicity in lymphoid malignant cell lines in a Cu-dependent manner both *in vitro* and *in vivo*. DS/Cu can significantly induce more apoptosis than DS or Cu alone *in vitro* while inhibiting the growth of xenograft lymphoma in mice. Furthermore, there has been a long history of using Cu to treat cancer [13]. However, the intracellular transport of Cu remains to be a major hurdle for its clinical use. N,N-diethyldithiocarbamte (deDTC), one of the DS derivatives, could bind to Cu forming a Cu (deDTC)_2_ complex which improves the intracellular trafficking of Cu [5]. Previous publication has already reported that children bearing lymphoma have higher serum Cu level than healthy ones. The Cu concentrations in the serum are associated with the disease stages [14]. Herein, DS can target cancer cells to treat lymphoma with little harm to normal tissues.

However, the mechanism of DS/Cu induced apoptosis remains unclear. Previous publications demonstrate that in combination with Cu, DS induces ROS activity in cancer cell lines [11]. Cancer cells are under higher ROS stress than normal tissues since they usually possess high proliferative rate [2]. However, high levels of ROS can damage DNA, mitochondrial inner membrane and membrane phospholipids leading to apoptosis [2]. Thus, generation of ROS might be a novel method to treat cancers. Recent studies have also demonstrated that generation of ROS by drugs can induce apoptosis in lymphoid malignant cell lines [15]. In consistence with these results, our study confirmed that DS/Cu induces ROS in Raji cell lines which might contribute to DS/Cu induced apoptosis. When ROS inhibitor NAC was added, the cytotoxicity of DS/Cu to Raji cell lines was significantly alleviated.

The c-Jun N-terminal kinase (JNK) signaling pathway is known to play a critical role in diverse cellular processes including regulation of proliferation, differentiation and apoptosis [9]. Moreover, previous studies indicated that the sustained activation of JNK is essential for drug-induced apoptosis in lymphoma cell lines *in vitro*[16]. Furthermore, ROS are potent activators of JNK through oxidative inactivation of endogenous JNK inhibitors, such as JNK phosphatases and glutathione *S*-transferase π [4]. In accordance with previous studies, we found that JNK pathway was persistently (over 24 h) activated (phosphorylation of cJun) by DS/Cu both *in vitro* and *in vivo* and blocked by ROS inhibitor NAC *in vitro*, confirming the important role of ROS in DS/Cu induced apoptosis.

As a double-edged sword, apart from induction of apoptotic factors, ROS also induces expression of anti-apoptotic factors. The effect of ROS on cancer cells depends on the balance between ROS-induced pro- and anti-apoptotic factors. Lymphoid malignant cell lines constitutively express high levels of NF-κB, an important anti-apoptotic factor [16]. However, previous study has demonstrated that certain NF-κB-regulated genes play a major role in regulating the amount of ROS in the cells [16]. ROS have various inhibitory or stimulatory roles in NF-κB signaling [11]. Owing to the sustained ROS generation by DS/Cu in Raji cells, p65, the vital component of NF-κB, was significantly inhibited by DS/Cu *in vitro* and *in vivo*, and this inhibition can be blocked by NAC.

Nrf2 is another important anti-apoptotic factor induced by ROS [3]. Nrf2 is a transcription factor that plays a vital role in activating antioxidant response which abolishes ROS activity, detoxifies harmful chemicals and eventually protects cancer cells from chemo- and radiotherapy-induced damage [3]. It can regulate various downstream genes with a wide variety of functions, such as cellular redox homeostasis, cell growth and apoptosis, DNA repair, inflammatory response, and the ubiquitin-mediated degradation pathway [3]. It has already been demonstrated that high background nuclear levels of Nrf2 in leukemia cells reduce the sensitivity of cancer cells to proteasome inhibitors [14]. Thus targeting Nrf2 might be an effective way to induce cancer cell apoptosis. Our study showed that DS/Cu induces ROS generation while inhibiting Nrf2 level in lymphoma Raji cells. Figure 4A showed that Nrf2 level might increase at low levels of ROS activity but decrease when ROS activity increases. We also observed the decrease of Nrf2 *in vivo*. Therefore, increase in ROS activity might cause the level of Nfr2 to decrease and thus lose its ability to protect lymphoma cells from oxidative stress and thus induce cell apoptosis. This phenomenon might due to the destruction effect of ROS on cells. Since cells from normal tissue usually have lower basal ROS level and complete protective mechanism, therefore the level of ROS will remain relatively low and high levels of Nrf2 will prevent the cells from ROS induced damage.

## Conclusion

Our study suggests that DS/Cu complex induced apoptosis in lymphoid malignant cells both *in vitro* and *in vivo*. Generation of ROS might be the core step in DS/Cu induced apoptosis. Moreover, ROS-related activation of JNK pathway as well as inhibition of NF-κB and Nrf2 might also contribute to the induced apoptosis.

## Abbreviations

DS: Disulfiram; Cu: Copper; ROS: Reactive Oxygen Species; NAC: N-acetyl-L-cysteine; MAPK: Mitogen activated protein kinase; JNK: C-Jun NH2-terminal kinase.

## Competing interests

The authors declare that they have no competing interests.

## Authors’ contributions

BX and WGW designed the experiments and wrote the manuscript. FLC performed the MTT assay and participated in the writing process. HJD did the flow cytometric analysis and participated in the writing process. PCHSH did the western blot assay. YY,Zh did the ROS assay, RWL did the statistical analysis, SYW ,PL, YY and JZ performed animal experiments and wrote the manuscript. All the authors have read and approved the manuscript.
